# Induced Pluripotency of Human Prostatic Epithelial Cells

**DOI:** 10.1371/journal.pone.0064503

**Published:** 2013-05-22

**Authors:** Hongjuan Zhao, Ning Sun, Sarah R. Young, Rosalie Nolley, Jennifer Santos, Joseph C. Wu, Donna M. Peehl

**Affiliations:** 1 Department of Urology, Stanford University School of Medicine, Stanford, California, United States of America; 2 Department of Medicine, Division of Cardiology, Stanford University School of Medicine, Stanford, California, United States of America; The Chinese University of Hong Kong, China

## Abstract

Induced pluripotent stem (iPS) cells are a valuable resource for discovery of epigenetic changes critical to cell type-specific differentiation. Although iPS cells have been generated from other terminally differentiated cells, the reprogramming of normal adult human basal prostatic epithelial (E-PZ) cells to a pluripotent state has not been reported. Here, we attempted to reprogram E-PZ cells by forced expression of Oct4, Sox2, c-Myc, and Klf4 using lentiviral vectors and obtained embryonic stem cell (ESC)-like colonies at a frequency of 0.01%. These E-PZ-iPS-like cells with normal karyotype gained expression of pluripotent genes typical of iPS cells (Tra-1-81, SSEA-3, Nanog, Sox2, and Oct4) and lost gene expression characteristic of basal prostatic epithelial cells (CK5, CK14, and p63). E-PZ-iPS-like cells demonstrated pluripotency by differentiating into ectodermal, mesodermal, and endodermal cells in vitro, although lack of teratoma formation in vivo and incomplete demethylation of pluripotency genes suggested only partial reprogramming. Importantly, E-PZ-iPS-like cells re-expressed basal epithelial cell markers (CD44, p63, MAO-A) in response to prostate-specific medium in spheroid culture. Androgen induced expression of androgen receptor (AR), and co-culture with rat urogenital sinus further induced expression of prostate-specific antigen (PSA), a hallmark of secretory cells, suggesting that E-PZ-iPS-like cells have the capacity to differentiate into prostatic basal and secretory epithelial cells. Finally, when injected into mice, E-PZ-iPS-like cells expressed basal epithelial cell markers including CD44 and p63. When co-injected with rat urogenital mesenchyme, E-PZ-iPS-like cells expressed AR and expression of p63 and CD44 was repressed. DNA methylation profiling identified epigenetic changes in key pathways and genes involved in prostatic differentiation as E-PZ-iPS-like cells converted to differentiated AR- and PSA-expressing cells. Our results suggest that iPS-like cells derived from prostatic epithelial cells are pluripotent and capable of prostatic differentiation; therefore, provide a novel model for investigating epigenetic changes involved in prostate cell lineage specification.

## Introduction

Induced pluripotent stem (iPS) cells generated by forced expression of certain transcription factors including Oct4, Klf4, c-Myc, and Sox2 resemble embryonic stem cells (ESCs) in morphology, gene expression, and ability to differentiate into any somatic cell type [1]. Because these cells, like ESCs, have enormous potential for cell therapy, drug screening and disease modeling, much effort has been invested in generating iPS cells from relevant cell types. To date, iPS cells have been derived from hepatocytes and stomach cells [2], pancreatic islet beta cells [3], lymphocytes [4], keratinocytes [5], peripheral blood cells [6], platelets [7], astrocytes [8], neural progenitor cells [9], endometrial cells [10], and adipose-derived stromal cells [11]. In addition, ESC-like cells have recently been derived from prostate cancer-associated stromal cells [12]. Generation of iPS cells from adult normal human epithelial cells has been achieved using retinal pigment epithelial cells and corneal limbal epithelial cells [13]. No attempt to derive iPS cells from normal human prostatic epithelial cells has been reported.

iPS cells provide a valuable resource for identifying epigenetic changes that occur during cell differentiation because reprogramming reverses the process of cell specification through epigenetic modification, erasing tissue-specific DNA methylation and re-establishing the embryonic-like methylome [14], [15]. iPS cells can then be re-differentiated into the desired cell type by appropriate inductive factors, and the epigenetic changes occurring throughout the differentiation process may be captured by temporal characterization of the epigenome as reported in ESC differentiation [16]. This process may be facilitated by the recently discovered epigenetic memory of iPS cells. Specifically, human iPS cells generated from hepatocytes (representative of endoderm), skin fibroblasts (mesoderm), and melanocytes (ectoderm) all retained a transcriptional memory of the original cells, which was partially explained by incomplete promoter DNA methylation [17]. In fact, both mouse and human iPS cells retain a residual DNA methylation pattern of the original somatic cells [18], [19]. This epigenetic memory biases the differentiation potential of iPS cells toward lineages related to the cell of origin [19], [20].

Little is known about the epigenetic changes underlying prostate differentiation, partly because of the lack of suitable models. While cell cultures have been a valuable resource for discovery of epigenetic changes occurring during differentiation, these are largely limited to tumor cell lines or transformed derivatives that carry genetic and epigenetic artifacts of accommodation to cell culture [21], [22]. Primary cultures are a more realistic model but have a limited life span [23]. If iPS cells could be generated from prostatic epithelial cells with defined factors, they would provide a tractable method for establishing immortal cultures of pluripotent cells from a single differentiated prostatic epithelial cell. Because the epigenetic changes that occurred during prostate differentiation will be erased in these iPS cells, these changes can be identified by comparing the epigenome of prostate epithelial cell-derived iPS cells and their differentiated progenies.

The prostatic epithelium is composed of two compartments of basal and luminal (secretory) epithelial cells. The lineage relationship between these two types of cells is controversial. While most studies suggest that prostate stem cells exist in the basal epithelial compartment and give rise to both basal and secretory cells [24], [25], several recent reports suggest that stem cells also exist in the secretory epithelial cell compartment [26], [27], suggesting that both basal and luminal epithelial cells are self-sustained lineages in the prostate [28]. In this study, we addressed two questions: whether prostatic basal epithelial cells could be reprogrammed to a pluripotent state by introducing Oct4, Sox2, Klf4, and c-Myc, and whether the resultant pluripotent cells could be induced to re-differentiate into basal and/or secretory prostatic epithelial cells. We demonstrate reprogramming of prostatic basal epithelial cells to iPS-like cells that are capable of differentiation into the three germ line lineages *in vitro*. Moreover, they are capable of differentiation into basal as well as secretory prostatic epithelial cells, establishing a novel model for identification of the molecular processes regulating prostatic cell lineages. Finally, we carried out a comprehensive DNA methylation profiling of iPS-like cells derived from prostatic basal cells at different time points of induced differentiation toward prostatic secretory cells and identified key pathways and regulators that may play important roles in prostatic differentiation.

## Materials and Methods

### Ethics statement

All animal studies were approved by the Stanford Administrative Panel on Laboratory Animal Care (APLAC) and done in compliance with the regulations for animal studies at Stanford University. Primary cultures of normal human prostatic epithelial (E-PZ) cells were established from radical prostatectomy specimens obtained immediately after surgery under a protocol approved by the Stanford Institutional Review Board. The participants provided their written informed consent to participate in this study.

### Culture and maintenance of iPS cells

E-PZ cells were established and characterized as previously described [29]. At low cell density (<50% confluency), E-PZ cells were cultured in Complete MCDB 105 medium, while Complete PFMR-4A medium was used for cells at high density (>50% confluency) [29]. The synthetic androgen R1881 (Perkin Elmer, Waltham, MA) was prepared in ethanol at 10 µM. SB431542 (Stemgent, San Diego, CA) and PD0325901 (Stemgent) and Thiazovivin (BioVision, Mountain View, CA) were each prepared in DMSO at l0 mM. iPS cells were derived from E-PZ cells (E-PZ-iPS-like cells) in hESC growth medium mTeSR-1(Stemcell Technologies Inc., Vancouver, Canada) using MEF feeder layers (Applied Stem Cell Inc., Menlo Park, CA) and maintained either on MEF feeder layers (Applied Stem Cell Inc.) or on Matrigel-coated tissue culture dishes (ES qualified; BD Biosciences, Bedford, MA) in DMEM/F12 (1∶1) medium supplemented with 5 ng/ml basic fibroblast growth factor (bFGF) (PeproTech, Rocky Hill, NJ), 10 ng/ml leukemia inhibitory factor (LIF) (Santa Cruz Biotechnology Inc., Santa Cruz, CA), and 5% knockout fetal bovine serum (FBS) (Invitrogen, Carlsbad, CA). E-PZ-iPS-like cells at passages 4–7 were used for analyses.

### Lentivirus production and transduction

293FT cells (Invitrogen) were inoculated at ∼80% confluency on 100-mm dishes in DME medium supplemented with 10% FBS and transfected with 12 µg of each lentiviral vector (Oct4, Sox2, Klf4, c-Myc) plus 8 µg packaging plasmids and 4 µg VSVG plasmids using Lipofectamine 2000 (Invitrogen) following the manufacturer's instructions. The cell culture medium was collected 48 h after transfection, filtered through a 0.45-µm pore-size cellulose acetate filter (Whatman, Piscataway, NJ), and mixed with PEG-it Virus Concentration Solution (System Biosciences, Mountain View, CA) overnight at 4°C. Viruses were precipitated at 1,500 g the next day and resuspended in Opti-MEM medium (Invitrogen).

### Immunofluorescence staining

Cells/spheres were fixed with 2% paraformaldehyde in phosphate-buffered saline (PBS) for 2 min, permeabilized with 0.5% Triton X-100 in PBS for 10 min, and blocked with 10% horse serum in PBS for 1 h. Cells were then stained with appropriate primary antibodies and AlexaFluor-conjugated secondary antibodies (Invitrogen). All antibodies are listed in Table S1.

### Immunohistochemistry

Mice kidneys carrying iPS cell grafts were fixed in 10% buffered formalin overnight and embedded in paraffin. Five-micron sections were cut from the blocks. Immunohistochemistry was performed as previously described [30]. Briefly, antigen retrieval was performed for 20 minutes using a citrate buffer (pH 6.0) following deparaffinization. After blocking endogenous peroxidase activity and non-specific binding using 0.3% hydrogen peroxide and 10% horse serum, respectively, the tissues were incubated overnight at 4°C with primary antibodies. The next day, slides were incubated with a biotinylated secondary antibody at room temperature for 30 minutes followed by another 30-minute incubation in peroxidase-conjugated streptavidin. Color was developed with 3,3-diaminobenzidine (DakoCytomation California, Inc., Carpinteria, CA). Counter-staining was performed with hematoxylin. All antibodies are listed in Table S1.

### Quantitative real-time polymerase chain reaction (qRT-PCR)

Total RNA from E-PZ and E-PZ-iPS-like cells was isolated using Trizol (Invitrogen) and reverse transcribed using SuperScript™ III Reverse Transcriptase (Invitrogen) according to the manufacturer's instructions. cDNA product was then mixed with SYBR® GreenER™ qPCR super mix (Invitrogen) and primers of interest in the subsequent PCR using a M×3005P® QPCR System (Strategene, La Jolla, CA). Transcript levels in each sample were determined in triplicate to minimize the experimental variation (standard deviation was calculated for each reaction). The transcript level of TATA box binding protein (TBP) was assayed simultaneously as an internal control. The primer sequences used in this study are listed in Table S2.

### Bisulfite pyrosequencing

Genomic DNA was isolated from E-PZ and E-PZ-iPS-like cells using AllPrep DNA/RNA mini kit (Qiagen, Germantown, MD) and submitted to EpigenDx Inc. (Hopkinton, MA) for bisulfite modification, PCR reactions, and pyrosequencing analysis. For the Nanog promoter, the methylation status at six CpG sites that are 431 bp, 512 bp, 517 bp, 519 bp, 556 bp, and 565 bp upstream of ATG was examined. For the Oct4 promoter, the methylation status at six CpG sites that are 14 bp, 24 bp, and 50 bp upstream of ATG and 80 bp, 97 bp, 70 bp, and 5 bp downstream of ATG was examined.

### Karyotyping by metaphase chromosome counting

Colcemid (Sigma-Aldrich, St. Louis, MO) was added to the culture medium at a final concentration of 1 ug/ml and cells were incubated at 37°C for 1 hour. Cells were then detached and resuspended in 5 ml of ice cold 0.56% KCl solution. After incubation at room temperature (RT) for 6 minutes, cells were fixed in 5 ml of methanol: glacial acetic acid (3∶1) solution. Cells were then dropped on clean slides and air dried. Slides were then stained with Geimsa dye and chromosomes were counted under microscopy. Fifty chromosome spreads were counted per cell line.

### In vitro differentiation

The following inductive differentiation systems were used: 1) For neural induction, E-PZ-iPS-like cells cultured on Matrigel were digested with collagenase type IV (Invitrogen) and seeded at a density of 1.0×10^5^ cells/cm^2^ on a poly-HEMA-coated dish (BD Biosciences). After culture for 7 d in Neurobasal medium (Invitrogen) supplemented with 1× B-27 (Invitrogen), 30 ng/mL bFGF (PeproTech) and 30 ng/mL epidermal growth factor (PeproTech) to induce sphere formation, spheres were then transferred onto poly-L-lysine-coated dishes (BD Biosciences) and cultured for 10 d in α-MEM supplemented with 2% FBS, 25 ng/mL bFGF, and 25 ng/mL BDNF (Peprotech); 2) Adipocyte and osteocyte inductions were performed according to the Human Mesenchymal Stem Cell Functional Identification Kit (R&D Systems, Minneapolis, MN); 3) For hepatocyte induction, cells at a density of 2.0×10^4^ cells/cm^2^ were cultured on collagen-coated dishes for 14 d in DMEM supplemented with 10% FBS, 1× insulin-transferrin-selenium (Gibco, Grand Island, NY), 10 nM dexamethasone (Sigma-Aldrich), 100 ng/mL hepatocyte growth factor (PeproTech), and 50 ng/mL FGF-4 (R&D Systems); 4) For prostate sphere differentiation: E-PZ-iPS-like cells cultured on Matrigel were digested with collagenase type IV (Invitrogen) and transferred to ultra-low attachment plates (Corning Life Sciences, Tewksbury, MA) for suspension culture for 8 days in DMEM/F12 (1∶1) medium supplemented with 5 ng/ml bFGF (PeproTech), 10 ng/ml LIF (Santa Cruz Biotechnology Inc.), and 5% knockout FBS (Invitrogen). For basal epithelial cell differentiation, E-PZ-iPS-like spheres were then cultured in Complete PFMR-4A medium [29] for 3 days. To induce expression of androgen receptor (AR), E-PZ-iPS-like spheres were further cultured in Complete PFMR-4A medium with 10 nM R1881 for 3 days. To induce expression of prostate-specific antigen (PSA), E-PZ-iPS-like spheres were co-cultured with rat urogenital sinus (UGS) isolated from E17 rat embryos as previously described [31] in Complete PFMR-4A medium with 10 nM R1881 for 5 days. Differentiation of E-PZ-iPS-like cells into prostate cells was then detected with appropriate markers by immunofluorescence.

### In vivo differentiation

Rat urogenital mesenchymal (UGM) cells were isolated as previously described [31]. Bona fide iPS cells derived from adult human skin fibroblasts (F-iPS) in Dr. Wu's lab at Stanford University or E-PZ-iPS-like cells were harvested from Matrigel-coated culture dishes and 100,000 cells were injected under the renal capsule of male RAG2^−/−^γC^−/−^ mice with or without 250,000 UGM cells as previously described [32]. A total of 100 µl of cell suspension in Matrigel (1∶1, vol/vol) was injected per mouse. For mice carrying F-iPS or E-PZ-iPS-like cells combined with UGM, a 25-mg testosterone pellet with a release rate of 0.2 mg/day was inserted into a small incision made under the skin between the shoulder blades. After 6–8 weeks, kidneys carrying iPS cell grafts were dissected, and fixed with 10% formaldehyde in PBS. Paraffin-embedded tissue sections were then generated and stained with hematoxylin and eosin.

### DNA methylation profiling

Genomic DNA was isolated from E-PZ-iPS-like cells cultured in control or differentiation-inducing media as described above and submitted to the Stanford Functional Genomic Facility for DNA methylation profiling using Infinium HumanMethylation450 BeadChip containing >485,000 methylation sites at single-nucleotide resolution (Illumina, San Diego, CA). Beta values representing the methylation levels of individual sites were extracted using Illumina GenomeStudio software. Statistical analyses were performed using Excel. Raw data were deposited in Gene Expression Omnibus (GEO) with accession number GSE45469.

## Results

### Generation of iPS cells from primary prostatic epithelial (E-PZ) cells

We chose to reprogram two E-PZ cultures, E-PZ-1 and E-PZ-2, derived from normal peripheral zone prostatic tissues of two men aged 56- and 44-years old, respectively. These primary cultures are a mixture of basal and transit amplifying cells since they express basal epithelial cell markers including cytokeratin 14 (CK14), cytokeratin 5 (CK5) and p63, but not secretory epithelial cell markers such as androgen receptor (AR) and prostate-specific antigen (PSA) [33]. As is typical of primary epithelial cell cultures, E-PZ cells are proliferative as shown by strong PCNA expression, and a subset of cells express cytokeratin 18 (CK18), which may represent transit amplifying cells [33].

**Figure 1:**
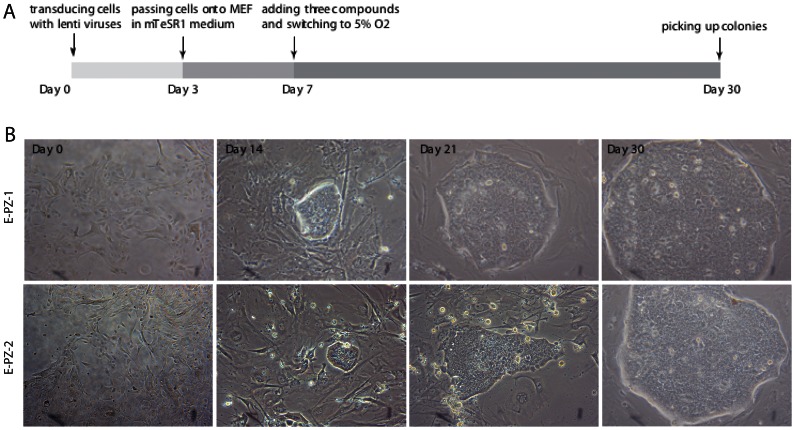
Generation of iPS cells from E-PZ cells by lentiviral transduction. (A) A diagram of the experimental design. The three compounds added to the medium at day 7 were 2 µM SB431542, 0.5 µM PD0325901, and 0.5 µM Thiazovivin. (B) Representative images of colonies derived from two E-PZ cultures at different time points.

E-PZ cells at passage 3 were transduced with individual lentiviruses containing human Oct4, Sox2, Klf4, and c-Myc at a 1∶1∶1∶1 ratio on day 0. On day 3 after the transduction, cells were transferred onto mouse embryonic fibroblast (MEF) feeder layers, with the culture medium switched from Complete MCDB 105 medium to human embryonic stem cell (ESC) growth medium mTeSR-1. To improve the efficiency of reprogramming, we cultured cells in low oxygen (5% O_2_) and in the presence of 2 µM SB431542, 0.5 µM PD0325901, and 0.5 µM Thiazovivin starting from day 7. Previous studies have shown that these three compounds and low oxygen enhance the efficiency of reprogramming [34], [35]. From day 14, clearly recognizable, tightly packed colonies with morphologies similar to ESCs appeared (Fig. 1). The number and size of the ESC-like colonies increased over time and large ESC-like colonies containing ≈400–500 cells could be isolated mechanically and transferred onto new feeder layers for further expansion by day 30 (Fig. 1). We designated these cells as E-PZ-iPS-like cells. We consistently observed ≈10 ESC-like colonies from 100,000 E-PZ cells, resulting in a reprogramming efficiency of ∼0.01%. This low reprogramming efficiency of E-PZ cells is consistent with and comparable to the results reported in previous studies using the Yamanaka four factors to reprogram human fibroblasts [36].

**Figure 2:**
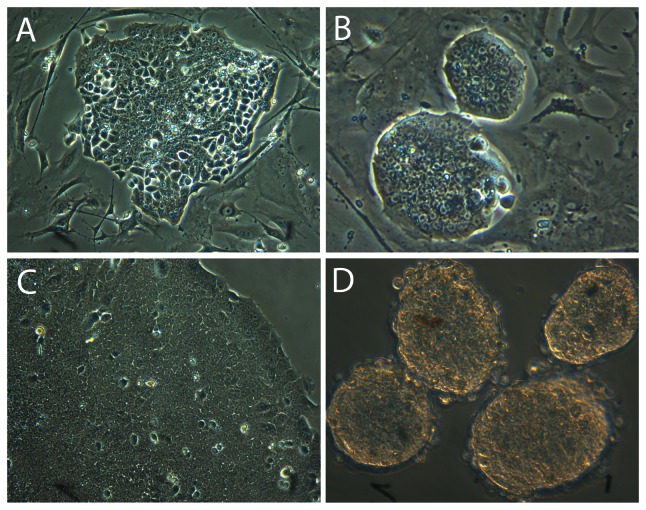
Optimization of culture conditions to maintain ESC-like morphology of E-PZ-iPS-like cells. Colonies lost ESC-like morphology in mTeSR-1 medium on feeder layers (A); however, they maintained ESC-like morphology in DMEM/F12 (1∶1) supplemented with 5 ng/ml bFGF, 10 ng/ml LIF and 5% knock out FBS on feeder layers (B) or Matrigel-coated plates (C). E-PZ-iPS-like cells formed spheres when cultured on ultra-low attachment plates in DMEM/F12 (1∶1) supplemented with 5 ng/ml bFGF, 10 ng/ml LIF and 5% knock out FBS (D).

We next attempted to expand the E-PZ-iPS-like cells that were isolated from the large ESC-like colonies in mTeSR-1 medium with feeder layers. We detached and broke the colonies into small pieces and transferred them onto new feeder layers. The cells attached and the colonies grew in size, but lost the ESC-like morphology over time. For example, the defined boundaries of the colonies became disrupted as the cells at the edge started to become loosely associated rather than tightly packed in the colonies (Fig. 2A). We therefore optimized the growth conditions by testing different types of media and found the best medium to be DMEM/F12 (1∶1) supplemented with 5 ng/ml bFGF, 10 ng/ml LIF, and 5% knock out FBS. In this medium, E-PZ-iPS-like colonies maintained their ESC-like morphology after multiple passages (Fig. 2B). Moreover, the ESC-like morphology was maintained when colonies were grown feeder-free on Matrigel-coated plates for generations in this medium (Fig. 2C). When cultured on ultra-low attachment plates, these cells form spheres in this medium (Fig. 2D).

### Characterization of E-PZ-iPS-like cells

**Figure 3:**
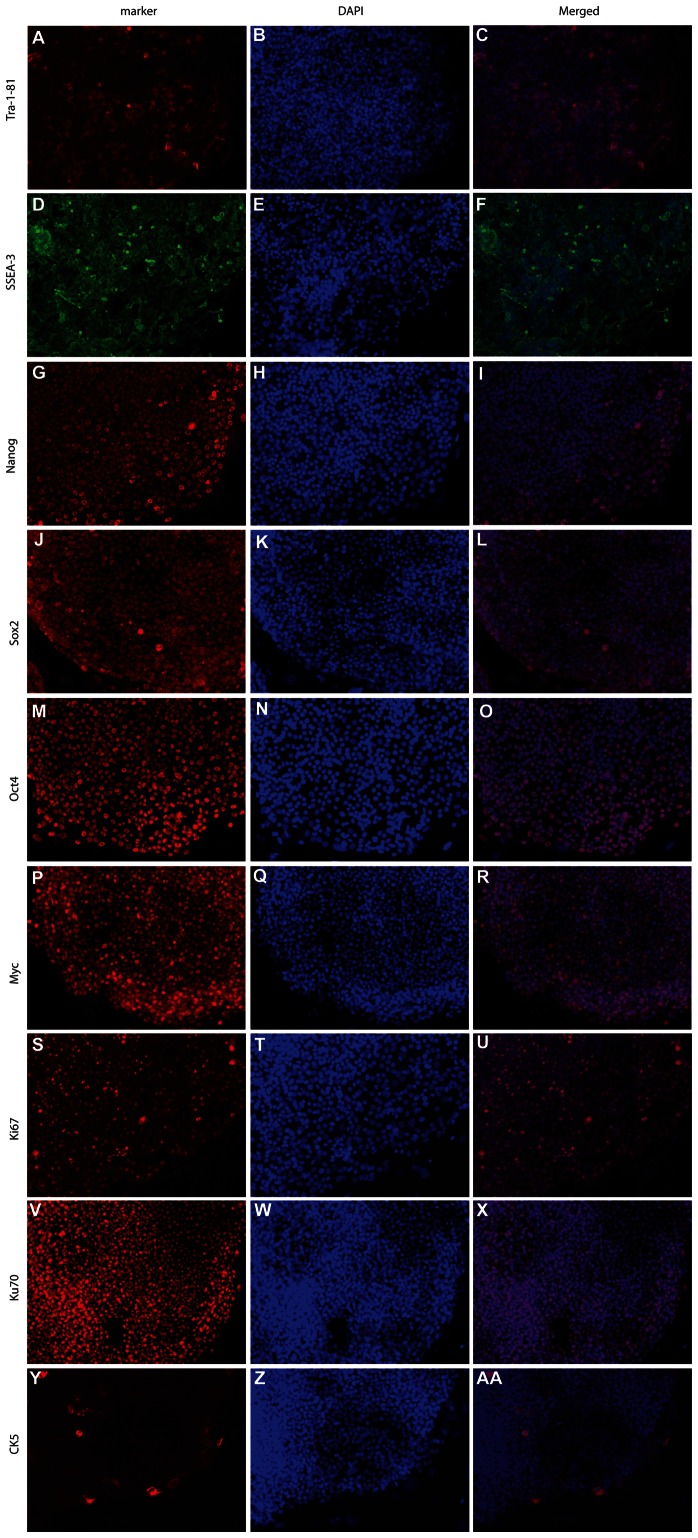
Immunofluorescence detection of marker expression in E-PZ-1-iPS-like-4 cells. E-PZ-1-iPS-like-4 cells showed strong cell membrane staining of TRA-1–81 (A) and SSEA-3 (D), and nuclear staining of Nanog (G), Sox2 (J), Oct4 (M), and c-Myc (P). These cells displayed strong nuclear staining of Ki67 (S) and the human nuclear antigen Ku70 (V). They did not express basal cell marker CK5 (Y) except in a few cells mostly located along the edge of the colonies. (B), (E), (H), (K), (N), (Q), (T), (W), and (Z) are DAPI staining of the nuclei. (C), (F), (I), (L), (O), (R), (U), (X), and (AA) are merged images of staining of DAPI and antibodies against specific markers.

We next characterized the E-PZ-iPS-like cells that were isolated from the large ESC-like colonies. We initially picked seven single colonies from reprogramming of E-PZ-1, and five from E-PZ-2. We characterized two cell lines from each individual in detail and obtained similar results from all 4 cell lines (E-PZ-1-iPS-like-4 and -7, E-PZ-2-iPS-like-1 and -5). Characteristics of clones 4 and 7, derived from E-PZ-1, are shown here as typical of the E-PZ-iPS-like cell lines. Immunofluorescence staining of E-PZ-1-iPS-like-4 cells with antibodies against TRA-1–81 (Fig. 3A–C), SSEA-3 (Fig. 3D–F), Nanog (Fig. 3G–I), Sox2 (Fig. 3J–L), Oct4 (Fig. 3M–O), and c-Myc (Fig. 3P–R) showed expression of these typical hESC markers. In addition, these cells displayed strong nuclear staining of Ki67 indicating that they are highly proliferative (Fig. 3S–U). Since the cell lines were generated in the presence of MEF, we confirmed the origin of these cells as human with an antibody specific for human nuclear antigen Ku70 (Fig. 3V–X). Similar expression of pluripotency genes was observed in E-PZ-2-iPS-like cells (Fig. S1). Moreover, we examined the expression of typical basal prostatic epithelial cell markers in E-PZ-iPS-like cells. Immunofluorescence staining did not detect expression of the basal cell marker CK5 (Fig. 3Y-AA) except in a few cells mostly located along the edge of the colonies. E-PZ-1-iPS-like-4 cells also did not express other basal cell markers, including CD44 and p63, nor secretory prostatic epithelial cell markers, including AR and PSA (Fig. S2). These results demonstrated a gain of pluripotent gene expression typical of iPS cells and a loss of gene expression characteristic of differentiated prostatic epithelial cells in E-PZ-iPS-like cells.

**Figure 4:**
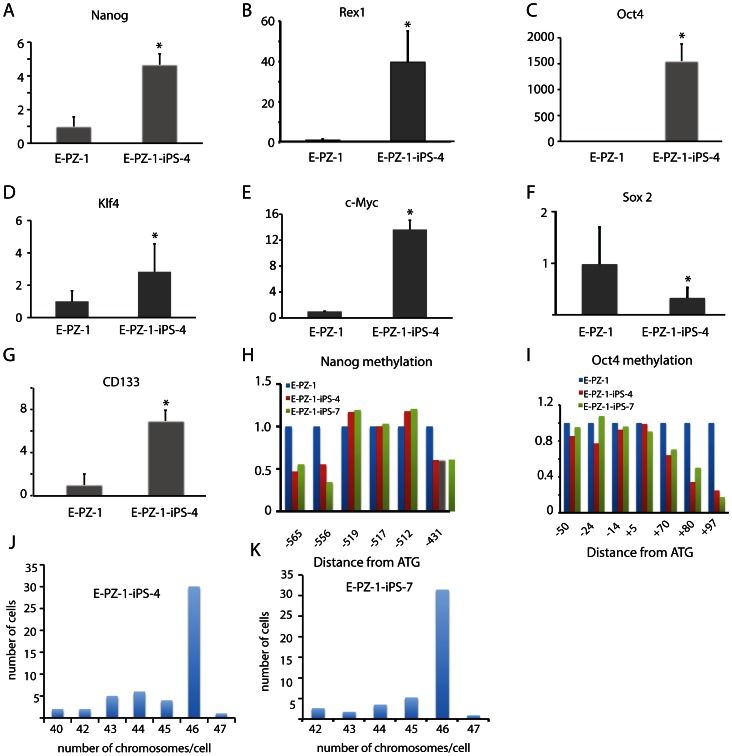
Determination of expression levels of pluripotent genes and methylation levels of their promoters in E-PZ-1 and E-PZ-1-iPS-like cells. mRNA levels of Nanog (A), Rex1 (B), Oct4 (C), Klf4 (D), c-Myc (E), Sox2 (F), and CD133 (G) were measured by qRT-PCR and normalized against TBP. Methylation of Nanog (H) and Oct4 (I) promoters were determined by bisulfite pyrosequencing. The Y-axis is the fold-level of gene expression or promoter methylation in E-PZ-1-iPS-like cells compared to those in E-PZ-1 cells, which were set as 1. (J) and (K) were histograms of the number of chromosomes in 100 E-PZ-1-iPS-4 and -7 cells, respectively, determined by metaphase chromosome counting.

The expression level of several pluripotency genes in E-PZ-iPS-like cells was also analyzed by qRT-PCR (Fig. 4). In E-PZ-1-iPS-like-4 cells, Nanog and Rex1 expression was increased ∼5- and ∼40-fold over that in E-PZ-1 cells, respectively (Fig. 4A–B). The Oct4 expression level was increased more than 1,500-fold compared to parent E-PZ-1 cells (Fig. 4C). Similarly, Klf4 and c-Myc expression was increased 2- and 13-fold in E-PZ-1-iPS-like-4 cells, respectively (Fig. 4D–E). Interestingly, Sox2 expression was significantly decreased (Fig. 4F) in E-PZ-1-iPS-like-4 cells. Finally, the expression of a prostate stem cell marker, CD133 [37], was increased ∼7-fold in E-PZ-1-iPS-like-4 cells compared to that in E-PZ-1 cells (Fig. 4G). Compared to human ESCs (line H9), Nanog, Rex1 and Oct4 expression was significantly lower and Klf4 and c-Myc expression was significantly higher in E-PZ-1-iPS-like-4 cells (Figure S3). Using primers specific to endogenous copies of the genes, we found that 99% of the total Oct4 expression in E-PZ-1-iPS-like-4 cells was from the endogenous copy of the gene, and 80% and 12% of total Klf4 and c-Myc expression were from the endogenous copies of the genes, respectively (Figure S3). Similar results were obtained from E-PZ-2-iPS-like cells (Figure S4). Overall, our results indicate that the expression of pluripotency genes in E-PZ-iPS-like cells was upregulated partly through activation of the endogenous copies of the genes.

The promoter regions of pluripotency genes in reprogrammed somatic cells are often demethylated, causing increased expression of downstream genes. We determined the methylation level of the promoter regions of Nanog and Oct4 in E-PZ-1 and E-PZ-1-iPS-like-4 and -7 cells by quantitative bisulfite pyrosequencing. Of the 6 CpG sites examined in the Nanog promoter, 3 showed demethylation in E-PZ-1-iPS-like cells compared to parent cells, while the other 3 didn't show significant changes in methylation (Fig. 4H). For the Oct4 promoter, 3 of 7 CpG sites showed demethylation while the other 4 did not (Fig. 4I). Chromosome spread counting demonstrated a normal karyotype, i.e. diploid, of E-PZ-1-iPS-1 and -7 cells (Fig. 4J–K). These results demonstrate epigenetic remodeling of the Oct4 and Nanog promoters in the E-PZ-iPS-like cells and are indicative of partial reprogramming.

### In vitro differentiation of E-PZ-iPS-like cells

**Figure 5:**
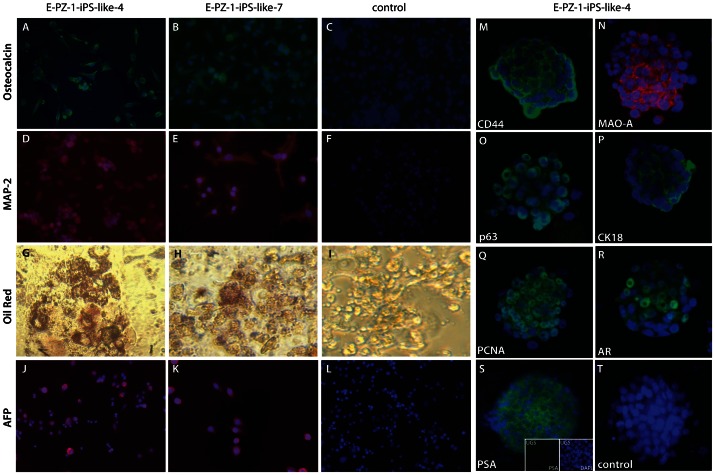
In vitro differentiation of E-PZ-1-iPS-like cells. E-PZ-1-iPS-like-4 and -7 cells were subjected to conditions that induced differentiation into neural cells (ectoderm), adipocytes (mesoderm), osteoblasts (mesoderm), or hepatocytes (endoderm). Osteoblast induction produced cells positive for osteocalcin (A and B). Neural induction generated cells positive for the neural cell marker MAP-2 (D and E). Adipocyte induction produced cells with lipid droplets that stained with oil red O (G and H). Hepatocyte induction generated cells positive for human α-fetoprotein (α-FP) (J and K). (C), (F) and (L) are negative controls that were stained with secondary antibodies only. (I) is a negative control without oil red staining. Some spheres derived from E-PZ-iPS-like cells and cultured in E-PZ medium expressed basal prostatic epithelial cell markers including CD44 (M), MAO-A (N), and p63 (O). In addition, some spheres expressed CK18 (P) and PCNA (Q). The spheres also expressed AR (R) in the presence of R1881. When co-cultured with rat UGS, a subset of the spheres expressed PSA (S, inserts showing UGS negative for PSA). In the absence of UGS, no PSA expression was detected (T).

To test the pluripotency of E-PZ-iPS-like cells, we first performed in vitro differentiation assays. Two individual lines of E-PZ-1-iPS-like cells each exhibited the capability of differentiating into derivatives of the three embryonic germ layers in vitro when subjected to conditions that induced differentiation into neural cells (ectoderm), adipocytes (mesoderm), osteoblasts (mesoderm), or hepatocytes (endoderm) (Fig. 5), as did F-iPS cells (data not shown). Osteoblast induction produced cells positive for osteocalcin in both E-PZ-1-iPS-like-4 and -7 cells (Fig. 5A–C). Neural induction generated cells positive for the neural cell marker MAP-2 in both cell lines (Fig. 5D–F). Adipocyte induction produced cells with lipid droplets that stained with oil red O (Fig. 5G–I). Hepatocyte induction generated cells positive for human α-fetoprotein (α-FP) (Fig. 5J–L). These results demonstrate that E-PZ-iPS-like cells can be directed to differentiate into ectodermal, mesodermal, and endodermal cells under the control of specific induction systems.

We also determined whether E-PZ-iPS-like cells could be directed to differentiate into prostatic epithelial cells in vitro. Spheres derived from E-PZ-1-iPS-like-4 cells and cultured in Complete PFMR-4A medium expressed basal prostatic epithelial cell markers including CD44 (Fig. 5M), MAO-A (Fig. 5N), and p63 (Fig. 5O). In contrast, F-iPS-derived spheres did not express these markers (Fig. S5). In addition, some spheres derived from E-PZ-1-iPS-like-4 cells expressed CK18 (Fig. 5P) and PCNA (Fig. 5Q), indicating a transit amplifying cell phenotype. Interestingly, spheres derived from E-PZ-1-iPS-like-4 cells and parental E-PZ-1 cells expressed a low level of AR in the presence of R1881, a synthetic androgen (Fig. 5R and Fig. S5). Moreover, when co-cultured with rat UGS, which has been shown to induce prostatic differentiation [31], a subset of the spheres derived from E-PZ-iPS-like cells expressed PSA (Fig. 5S, inserts showing UGS negative for PSA), while spheres cultured in the absence of UGS were negative for PSA (Fig. 5T). In contrast, neither F-iPS cells nor E-PZ-1 cells expressed PSA when cultured under the same conditions (Fig. S5). This prostatic differentiation capability was verified in E-PZ-2-iPS-like cells (Fig. S6). These results suggest that E-PZ-iPS-like cells have the capacity to differentiate into prostatic basal, transit amplifying and secretory epithelial cells.

### In vivo differentiation of E-PZ-iPS-like cells

**Figure 6:**
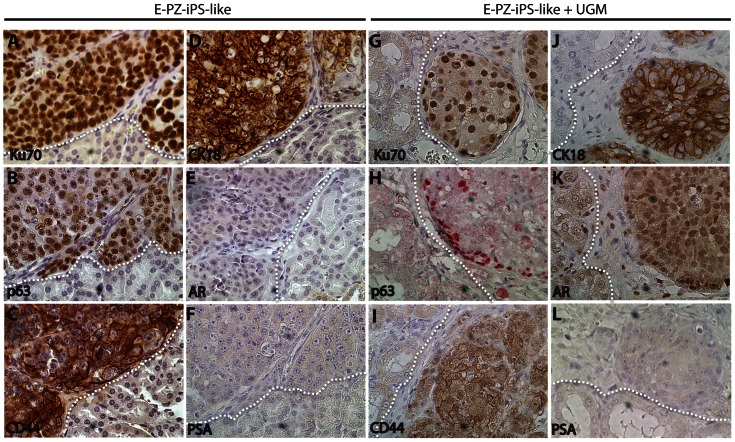
In vivo differentiation of E-PZ-1-iPS-like cells. E-PZ-1-iPS-like cells injected under the renal capsule of immunodeficient mice expressed human-specific nuclear antigen Ku70 (A) and basal prostatic epithelial markers including p63 (B) and CD44 (C). A subset of cells was positive for the transit amplifying epithelial cell marker CK18 (D) but not the secretory cell markers AR (E) or PSA (F). When combined with UGM, E-PZ-1-iPS-like cells gave rise to tissue expressing Ku70 (G) and CK18 (J). In addition, p63 was expressed only by cells at the edge of the tissue (H) and CD44 expression was reduced (I). Although the cells were negative for PSA (L), they expressed an intermediate level of AR in the nuclei (K). White dotted lines mark the boundary of grafts derived from E-PZ-1-iPS-like cells and mouse kidney. All magnifications are 40×.

To determine the differentiation potential of E-PZ-iPS-like cells in vivo, we injected E-PZ-iPS-like cells under the renal capsule of immunodeficient mice. After 6–8 weeks, tissue masses formed under the renal capsule with 100% frequency. We did not observe teratoma-like histology in the tissue masses derived from E-PZ-iPS-like cells. The cells expressed human-specific nuclear antigen Ku70 (Fig. 6A), demonstrating their human origin. In addition, they expressed basal prostatic epithelial markers including p63 (Fig. 6B) and CD44 (Fig. 6C). A subset of cells were positive for the transit amplifying/luminal epithelial cell marker CK18 (Fig. 6D), but not for AR (Fig. 6E) or PSA (Fig. 6F). Similar results were obtained using E-PZ-2-iPS-like cells (Fig. S7). These results suggest that E-PZ-iPS-like cells retained the capability of differentiating into basal/transit amplifying cells in vivo.

We attempted to induce further differentiation of the E-PZ-iPS-like cells toward the secretory epithelial phenotype by combining the cells with rat urogenital mesenchymal (UGM) cells, which have been shown previously to induce prostatic differentiation in vivo [31]. The tissue masses formed under the renal capsule in the presence of UGM and androgen supplementation were smaller in size and showed different marker expression patterns compared to those in the absence of UGM and androgen. Specifically, the basal prostatic epithelial cell marker p63 was expressed only by the cells at the edge of the cell clusters (Fig. 6H) instead of all cells in a cluster (Fig. 6B). Expression of another basal prostatic epithelial cell marker, CD44 (Fig. 6I), was less intense than that in the absence of UGM and androgen (Fig. 6C). Moreover, although the cells were negative for PSA (Fig. 6L), they expressed an intermediate level of AR in the nucleus (Fig. 6K), whereas no AR expression was observed in the absence of UGM and androgen (Fig. 6E). These results suggest that in the presence of UGM and androgen, E-PZ-iPS-like cells were induced toward a more complete prostatic differentiation with cells displaying both basal and secretory epithelial characteristics.

**Figure 7:**
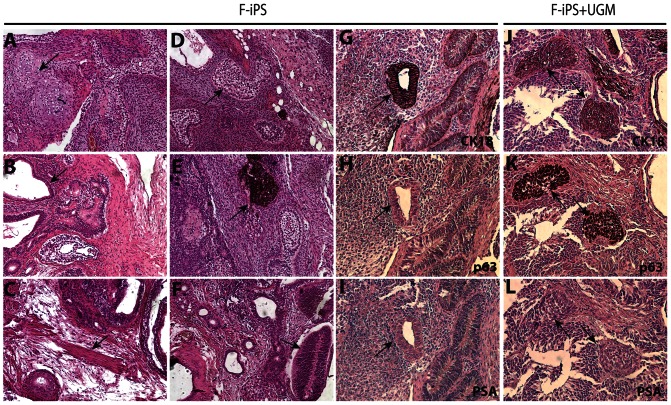
In vivo differentiation of F-iPS cells. F-iPS cells injected under the renal capsule of immunodeficient mice showed histological characteristics typical of teratoma including cartilage (A), gut-like epithelium (B), muscle (C), adipose tissue (D), pigmented cells (E), and neuroepithelial rosettes (F). Many glands were positive for CK18 (G), but not p63 (H) or PSA (I). When combined with UGM, F-iPS cells gave rise to cell clusters expressing both CK18 (J) and p63 (K), but not PSA (L). All magnifications are 20×.

As a comparison, we performed the same in vivo differentiation experiments using F-iPS cells. F-iPS cells without UGM injected under the renal capsule of immunodeficient mice formed teratomas 6–8 weeks after injection. Histological analysis showed the presence of cartilage (Fig. 7A), gut-like epithelium (Fig. 7B), muscle (Fig. 7C), adipose tissue (Fig. 7D), pigmented cells (Fig. 7E), and neuroepithelial rosettes (Fig. 7F). Many glandular structures were positive for CK18 (Fig. 7G), but negative for p63 and PSA (Fig. 7H–I). Tissues with similar histology were formed when F-iPS cells were combined with UGM. However, cell clusters that are positive for both CK18 and p63 (Fig. 7J and K) were observed, indicating a precursor phenotype of both basal and secretory epithelial characteristics. These cells were negative for PSA (Fig. 7L), confirming they are not mature secretory epithelial cells. These results suggest that F-iPS cells and E-PZ-like iPS cells were both capable of incomplete prostate differentiation in vivo.

### Identification of epigenetic changes during prostate differentiation

**Figure 8:**
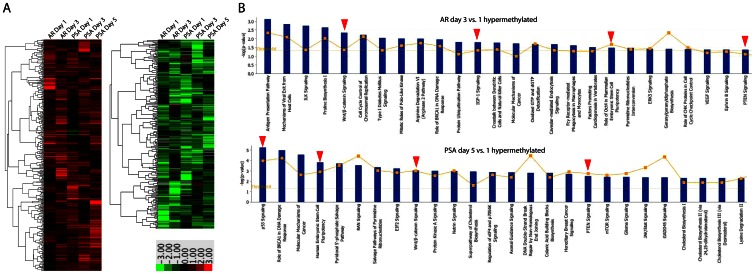
Identification of DNA methylation changes in prostatic differentiation using E-PZ-1-iPS-4 cells. (A) Genes that showed significantly higher (in red) or lower (in green) levels of methylation in cells cultured under AR- or PSA- inducing conditions compared to control across different time points of the induction process. (B) Canonical pathways identified by IPA that are enriched with genes hypermethylated in AR and PSA induced cells at late time points compared to early time point. Red arrows point out key pathways known to be involved in prostatic cell differentiation.

To demonstrate the utility of E-PZ-iPS-like cells as a model in elucidating the mechanisms of prostate differentiation, we analyzed temporal epigenetic changes occurring during the induction of secretory prostatic differentiation of E-PZ-iPS-like cells using DNA methylation profiling. DNA methylation levels of >485,000 sites were measured in spheres generated from E-PZ-1-iPS-like-4 cells cultured either in iPS cell medium as control, or in Complete PFMR-4A medium with 10 nM R1881 to induce AR expression for 1 or 3 days. In addition, spheres cultured in Complete PFMR-4A medium with 10 nM R1881 in the presence of UGS to induce PSA expression were harvested at 1, 3, or 5 days to capture methylation changes during mature secretory cell differentiation. In all, 5 pairs of samples were compared in the study, i.e., AR day 1 vs. control, AR day 3 vs. control, PSA day 1 vs. control, PSA day 3 vs. control, and PSA day 5 vs. control. Changes in methylation levels were examined in two ways. First, fold-change was calculated for each pair of samples as methylation level in induced cells divided by that in corresponding control cells. We focused on genes whose methylation levels increased or decreased by at least 50% in induced cells compare to corresponding control cells in at least 3 pairs of samples. Second, student's t-test was performed between the 5 control and 5 induced cells as two groups. Only genes with significant differential methylation levels in control vs. induced groups were selected. After filtering data with these two criteria, we identified 398 genes and 250 genes that were consistently and significantly hyper- or hypo-methylated in induced cells compared to control, respectively (Table S3). The fold-changes of these genes in induced vs. control cells are shown in Fig. 8A. Ingenuity Pathways Analysis (IPA) was performed to identify biological functions enriched for the 398 or 250 genes. For example, the top biological function in which the 398 hypermethylated genes in induced cells were enriched is embryonic development with >60 specific processes (Table S4). In particular, formation of prostatic bud was significantly affected (p = 0.0138) through increased methylation of BMP7 and Wnt5A. It has been shown that newborn rat prostate cultured with exogenous Wnt5a protein exhibited signs of delayed maturation and secretory cell differentiation [38], suggesting that Wnt5a inhibits secretory differentiation. This is consistent with increased methylation of Wnt5a, which has been shown to silence Wnt5a expression [39], [40], in the differentiation of E-PZ-iPS-like cells toward the prostatic secretory cell lineage.

We further compared methylation levels in cells cultured under AR induction conditions for 1 vs. 3 days, and PSA induction conditions for 1 vs. 5 days. Genes that showed >4-fold higher or lower methylation levels were selected for further analysis (Table S5). IPA analysis identified key canonical pathways and upstream regulators that have been shown to play important roles in prostatic differentiation (Fig. 8B and Table S6). As expected, genes functioning in human ESC pluripotency were significantly hypermethylated in AR- and PSA- induced cells at later time points compared to day 1 (Fig. 8B). Similarly, PTEN pathway components were significantly hypermethylated in both differentiation processes, consistent with the recent finding that conditional ablation of PTEN in prostate basal cells promotes basal-to-luminal differentiation in mice [41]. Moreover, Nkx3.1, the earliest known marker of prostate epithelium during embryogenesis, was identified as a significant upstream regulator of genes that were demethylated in cells cultured under PSA-inducing conditions for 5 days compared to those for only 1 day (Table S6), consistent with the finding that Nkx3.1 knock-out resulted in defects in prostate ductal morphogenesis and secretory protein production [42]. These results demonstrated that DNA methylation profiling using E-PZ-iPS-like cells undergoing differentiation captured epigenetic changes in key genes and pathways that are known to play important roles in prostatic differentiation.

## Discussion

We have generated iPS-like cells from human basal prostatic epithelial cells by forced expression of four reprogramming factors including Oct4, Sox2, Klf4 and c-Myc. These cells have several characteristics of iPS cells derived from other types of somatic cells, including: 1) expression of pluripotency markers such as Tra-81, Nanog, and SSEA-3 in vitro; 2) activation of the endogenous copy of pluripotent genes such as Oct4; 3) hypomethylation in the promoter regions of Oct4 and Nanog; and 4) differentiation into derivatives of the three embryonic germ layers in vitro under induction. However, they are different from iPS cells derived from other types of somatic cells such as F-iPS cells in several ways, including: 1) lack of formation of teratomas in vivo; 2) ability to differentiate into prostatic epithelial cells in vitro and in vivo; and 3) induction by UGM toward further prostatic differentiation in vivo. The differences are likely due to incomplete reprogramming of the epigenome, which has been observed in human iPS cells derived from a variety of cell types [43]. It is also worth noting that the E-PZ-iPS-like cells are diploid, suggesting that they have a stable genome and are different from immortalized cells generated by virus induction of EBV, HPV E6/7 and SV40 T Antigen that almost never have the normal diploid karyotype. Overall, our results demonstrate that iPS-like cells can be derived from normal adult human prostatic epithelial cells. These cells are pluripotent and capable of prostatic differentiation, and therefore may provide a useful tool to better understand the biology of prostatic differentiation.

Although in theory F-iPS cells should give rise to mature prostate glands as do ESC [44], it is not the case in our hands. In addition, E-PZ-iPS-like cells failed to express PSA in vivo in the presence of UGM for unknown reasons. One possibility is that the time of induction used in this study, i.e., 6–8 weeks, was not long enough for cells to fully mature and express PSA. In support of this possibility, Taylor et al. showed that prostate cells derived from human ESC combined with UGM expressed PSA after 8–12 weeks in vivo [44]. Similarly, mature secretory cells expressing PSA were observed in grafts derived from prostate stem cells combined with UGM after 8–24 weeks [45]. Alternatively, it is possible that in vitro induction conditions used in our study promoted secretory differentiation of E-PZ-iPS-like cells to a more complete stage than in vivo conditions for unknown reasons.

Differentiated somatic cells are replete with epigenetic regulatory devices that lock characteristic gene expression patterns into place. In general, such cells are quite refractory to reprogramming, as indicated by the very low efficiency of iPS generation, and lack of complete reprogramming to the ESC state. The reprogramming efficiency observed for E-PZ cells was similar to that reported for other terminally differentiated cells. Unlike previous studies reporting improved reprogramming efficiency when p53 expression is decreased or Glis1 expression is increased [46], [47], inhibition of p53 expression during reprogramming using lentiviruses carrying p53 shRNA or transducing cells with adenovirus containing Glis1 did not improve the reprogramming efficiency for E-PZ cells (unpublished data). It may be possible to increase the efficiency using other factors or improved methods since different factors may have different effects depending on the cell type being reprogrammed [48], [49].

Many somatic cells, including terminally differentiated cells, can be reprogrammed to an ESC-like state [50]. Our results demonstrated the capacity of E-PZ cells to be reprogrammed to a pluripotent state. However, this reprogramming was likely incomplete compared to fully reprogrammed iPS cells because the E-PZ-iPS-like cells did not have the same molecular profile as ESCs nor the same capacity to form teratomas in mice. We suspect that this is due to the imbalance of the expression levels of the four reprogramming factors. Previous studies support the hypothesis by Yamanaka et al. that reprogramming is a two-step process consisting of an initial transformation induced by c-Myc and Klf4 followed by a pluripotency induction process regulated by Oct4 and Sox 2 [51], [52]. Our E-PZ-iPS-like cells showed higher c-Myc and Klf4 expression, similar Oct4 expression, and lower Sox2 expression compared to H9 ESCs. Therefore, the initial transformation process was accomplished by sufficient c-Myc and Klf 4 expression and reflected by the immortal growth of the E-PZ-iPS-like cells. However, the induction of pluripotency was supported by adequate Oct4 but not Sox2 expression. This is consistent with previous findings that direct interactions between Klf4, Oct4, and Sox2 are critical for somatic cell reprogramming [53]. It would be interesting to determine whether small molecule inhibition of MAP kinase and glycogen synthase kinase 3 with LIF would help to achieve full reprogramming of E-PZ cells since this has been shown to promote transition to naive pluripotency in partially reprogrammed mouse cells [54]. Other small molecules such as 5-aza-2′-deoxycytidine, a DNA methylation inhibitor, may also enable a full reprogramming of E-PZ cells as observed in other terminally differentiated cell types [55].

E-PZ-iPS-like cells retained differentiation capability toward the parental cell lineage, i.e., prostatic epithelial cells, consistent with the theory of “epigenetic memory”. It has been shown that iPS cells derived by factor-based reprogramming of adult murine tissues harbor residual DNA methylation signatures characteristic of their somatic tissue of origin, which favors their differentiation along lineages related to the donor cell, while restricting alternative cell fates [19], [20]. In addition, reprogrammed human retinal pigmented epithelial (RPE) cells show a tendency for spontaneous redifferentiation into RPE cells, and human pancreatic islet beta cells are predisposed to differentiate more readily into insulin-producing cells, suggesting epigenetic imprints from the parental cells in the reprogrammed cells [3], [56]. Such an epigenetic memory of the donor tissue could be reset by differentiation and serial reprogramming, or by treatment of iPS cells with chromatin-modifying drugs [20]. It would be interesting to determine whether E-PZ-iPS-like cells would lose their prostate differentiation capability by such manipulations.

Perhaps the most interesting finding of our study is that E-PZ-iPS-like cells are capable of prostatic secretory differentiation in vitro. Molecular mechanisms and pathways involved in prostatic differentiation, especially secretory cell differentiation, are poorly understood due to the lack of suitable models. Current cell culture-based models only attain limited AR and PSA expression [57], [58]. The more reliable method to achieve secretory differentiation in human cells is tissue recombination, which relies on the induction of prostatic differentiation of human ESCs, human prostate stem/progenitor cells or immortalized human prostatic epithelial cell lines by rodent UGM and/or seminal vesicle mesenchyme in vivo [32], [44], [45], [59]. Although this method has led to important advances in our understanding of prostate biology during development and disease, in vivo studies are time- and resource- consuming. Thus, E-PZ-iPS-like cells may serve as a much-needed facile in vitro model to elucidate the molecular basis underlying prostatic differentiation because they are capable of differentiating into cells expressing both basal and secretory cell markers upon induction. The differentiation process can be repeated in a highly controlled manner to generate large numbers of cells for studies in vitro.

As proof-of-principle, we carried out a comprehensive epigenetic characterization of E-PZ-iPS-like cells at different time points of differentiation. DNA methylation profiling captured key genes and pathways that have been implicated in prostatic differentiation including Wnt5a, PTEN, and Nkx3.1. In addition, new genes and pathways have been selected as possible regulators of secretory differentiation. For example, many target genes of miRNAs let-7, mir-1, and mir-145 were hypermethylated in cells cultured under AR-inducing conditions for 3 days compared to 1 day (Table S6), suggesting a promoting role of these microRNAs in secretory differentiation of prostatic epithelial cells. Interestingly, a recent study demonstrated that loss of let-7 up-regulates EZH2 in prostate cancer with the acquisition of cancer stem cell signatures [60], suggesting that let-7 functions to promote cell differentiation through repression of EZH2 in prostate cancer. Our results indicate that let-7 may play a similar role in normal prostatic epithelial cells. Both mir-1 and mir-145, whose expression is downregulated in prostate cancer, have been implicated as pro-differentiation factors by silencing the stem cell self-renewal and pluripotency program or inhibiting epithelial-mesenchymal transition (EMT), respectively [61], [62]. Altogether, our study demonstrated that epigenetic changes identified using E-PZ-iPS-like cells as a model may serve as a valuable resource for dissecting the mechanisms of prostatic differentiation.

## Supporting Information

Figure S1 **Immunofluorescence detection of pluripotency gene expression in E-PZ-2-iPS-like-1 cells.** E-PZ-2-iPS-like-1 cells showed strong nuclear staining of c-Myc (**A**), Nanog (**D**), Oct4 (**G**), Sox2 (**J**), and membrane staining of Tra-1-81 (**M**). (**B**), (**E**), (**H**), (**K**) and (**N**) are DAPI staining of the nuclei of the same cells in (**A**), (**D**), (**G**), (**J**), and (**M**), respectively. (**C**), (**F**), (**I**), (**L**), and (**O**) are merged images of (**A**) and (**B**), (**D**) and (**E**), (**G**) and (**H**), (**J**) and (**K**), (**M**) and (**N**), respectively.
(TIF)

Figure S2 **Immunofluorescence detection of marker expression in E-PZ-1-iPS-like-4 cells.** E-PZ-1-iPS-like-4 cells showed strong nuclear staining of PCNA (**A**), which was used as a positive control. They did not express basal cell marker p63 (**B**), CD44 (**C**), AR (**D**), or PSA (**E**). (**F**), (**G**), (**H**), (**I**) and (**J**) are DAPI staining of the nuclei of the same cells in (**A**), (**B**), (**C**), (**D**), and (**E**), respectively.
(TIF)

Figure S3 **Comparison of expression levels of pluripotent genes in human ESCs (line H9) and E-PZ-1-iPS-like cells.** mRNA levels of Nanog (**A**), Rex1 (**B**), total and endogenous Oct4 (**C**), total and endogenous Klf4 (**D**), and total and endogenous c-Myc (**E**) were measured by qRT-PCR and normalized against TBP. The Y-axis is the fold-level of gene expression in E-PZ-1-iPS-like cells compared to those in ES cells, which were set as 1. Asterisks indicate statistical significance by t-test.
(TIF)

Figure S4 **Expression levels of pluripotent genes in E-PZ-2-iPS-like-1 cells.** mRNA levels of Nanog (**A**), total c-Myc (**B**), total Oct4 (**C**), CD133 (**D**), total Sox2 (**E**), and total Klf4 (**F**) in E-PZ-2-iPS-like-1 cells were compared to parent E-PZ-iPS-2 cells. Total and endogenous Klf4 (**G**), total and endogenous c-Myc (**H**), and total and endogenous Oct4 (**I**) were measured by qRT-PCR and normalized against TBP. In (**A**)–(**F**), the Y-axis is the fold-level of gene expression in E-PZ-2-iPS-like cells compared to those in E-PZ-2 cells, which were set as 1. In (**G**)–(**I**), the Y-axis is the fold-level of gene expression in E-PZ-2-iPS-like cells compared to those in ES cells, which were set as 1. Asterisks indicate statistical significance by t-test.
(TIF)

Figure S5 **In vitro differentiation of F-iPS and E-PZ cells.** F-iPS and E-PZ cells were subjected to conditions that induced differentiation of secretory prostatic epithelial cells, i.e., spheres were cultured in Complete PFMR-4A medium supplemented with 10 nM R1881 in the presence of rat UGS. An F-iPS-derived sphere showed strong staining of PCNA (**A**), but not CK14 (**B**), p63 (**C**), AR (**D**) or PSA (**E**). Spheres derived from E-PZ cells expressed PCNA (**K**). Some spheres also expressed an intermediate level of AR (**L**), but no PSA was detected (**M**). (**F**), (**G**), (**H**), (**I**), (**J**), (**N**), (**O**) and (**P**) are DAPI staining of the nuclei of the same cells in (**A**), (**B**), (**C**), (**D**), (**E**), (**K**), (**L**) and (**M**), respectively.
(TIF)

Figure S6 **In vitro differentiation of E-PZ-2-iPS-like-1 cells.** E-PZ-2-iPS-like-1 were cultured in E-PZ medium expressed basal prostatic epithelial cell markers including CD44 (**A**), MAO-A (**D**), and p63 (**G**). In addition, some spheres expressed CK18 (**J**) and AR (**M**) in the presence of R1881. When co-cultured with rat UGS, a subset of the spheres expressed PSA (**P**). (**B**), (**E**), (**H**), (**K**), (**N**) and (**Q**) are DAPI staining of the nuclei of the same cells in (**A**), (**D**), (**G**), (**J**), (**M**), and (**P**) respectively. (**C**), (**F**), (**I**), (**L**), (**O**), and (**R**) are merged images of (**A**) and (**B**), (**D**) and (**E**), (**G**) and (**H**), (**J**) and (**K**), (**M**) and (**N**), (**P**) ad (**Q**), respectively.
(TIF)

Figure S7 **In vivo differentiation of E-PZ-2-iPS-like-1 cells.** E-PZ-2-iPS-like-1 cells injected under the renal capsule of immunodeficient mice expressed basal prostatic epithelial markers p63 (**B**) and transit amplifying epithelial cell marker CK18 (**A**, but not the secretory cell markers AR (**C**) or PSA (**D**). When combined with UGM, E-PZ-2-iPS-like-1 cells gave rise to cell clusters that uniformly expressed CK18 (**E**), and p63 but only at the edge (**F**). Although the cells were negative for PSA (**H**), they expressed AR in the nuclei (**G**). White dotted lines mark the boundary of grafts derived from E-PZ-2-iPS-like-1 cells and mouse kidney. All magnifications are 20×.
(TIF)

Table S1 **Antibodies used in the study.**
(DOCX)

Table S2 **Primer sequences used in the study.**
(DOCX)

Table S3 **Genes that are hyper- or hypo-methylated across 5 pairs of samples at different time points of AR or PSA induction.**
(XLS)

Table S4 **IPA analysis identified embryonic development as the top biological function in which hypermethylated genes were enriched during AR and PSA induction.**
(XLS)

Table S5 **Genes whose methylation levels changed >4-fold in AR day 3 vs 1 and PSA day 5 vs 1 comparisons.**
(XLS)

Table S6 **Canonical pathways and upstream regulators identified by IPA using genes in Table S5.**
(XLS)
