# Supplementary figures and images for: Induced Pluripotency of Human Prostatic Epithelial Cells
Source: PLoS One. 2013 May 22;8(5):e64503. doi: 10.1371/journal.pone.0064503 (PMC3661502; doi:10.1371/journal.pone.0064503)

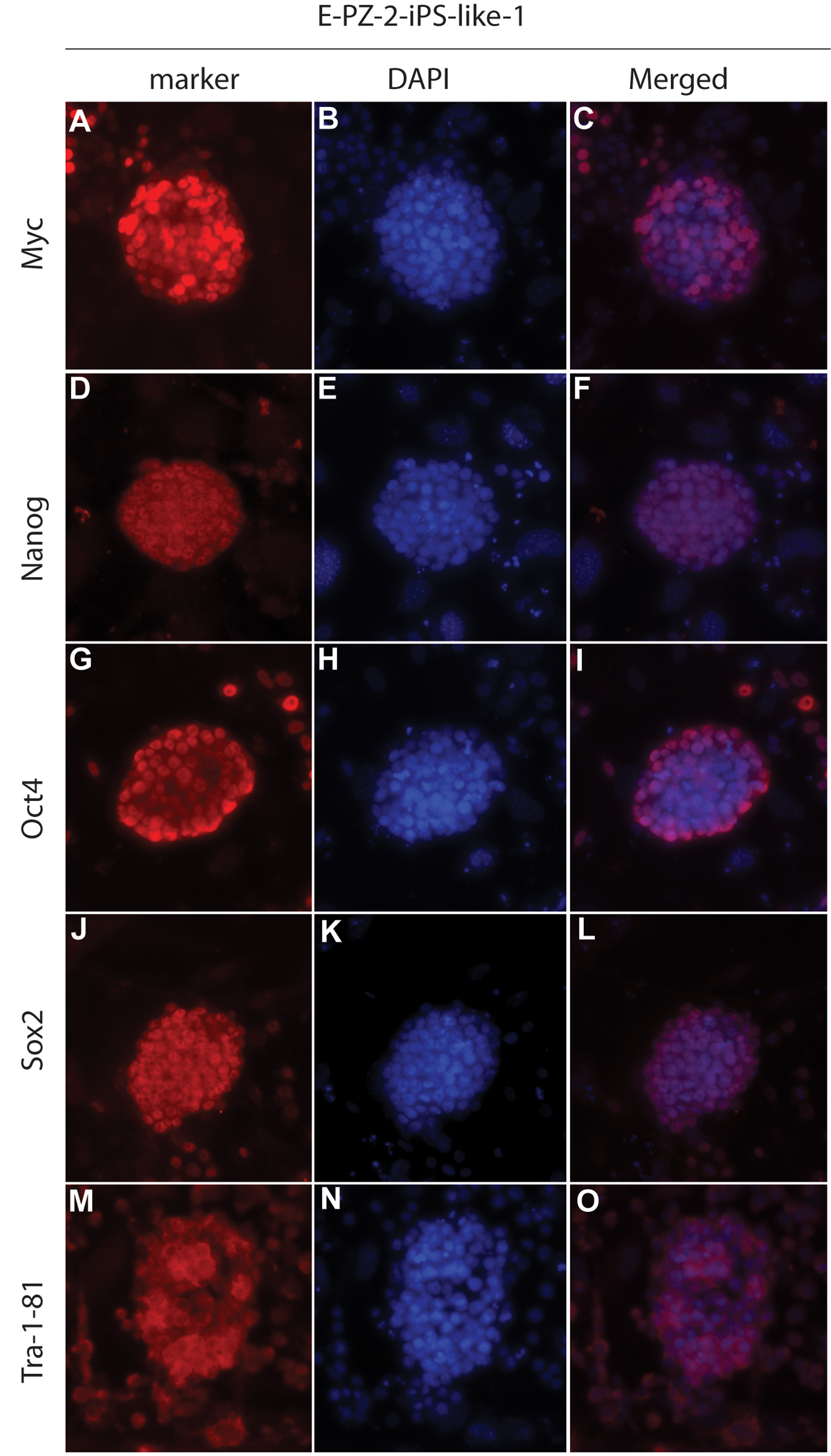

Supplement: Figure S1 — Immunofluorescence detection of pluripotency gene expression in E-PZ-2-iPS-like-1 cells. E-PZ-2-iPS-like-1 cells showed strong nuclear staining of c-Myc (A), Nanog (D), Oct4 (G), Sox2 (J), and membrane staining of Tra-1-81 (M). (B), (E), (H), (K) and (N) are DAPI staining of the nuclei of the same cells in (A), (D), (G), (J), and (M), respectively. (C), (F), (I), (L), and (O) are merged images of (A) and (B), (D) and (E), (G) and (H), (J) and (K), (M) and (N), respectively. (TIF) [file pone.0064503.s001.tif]

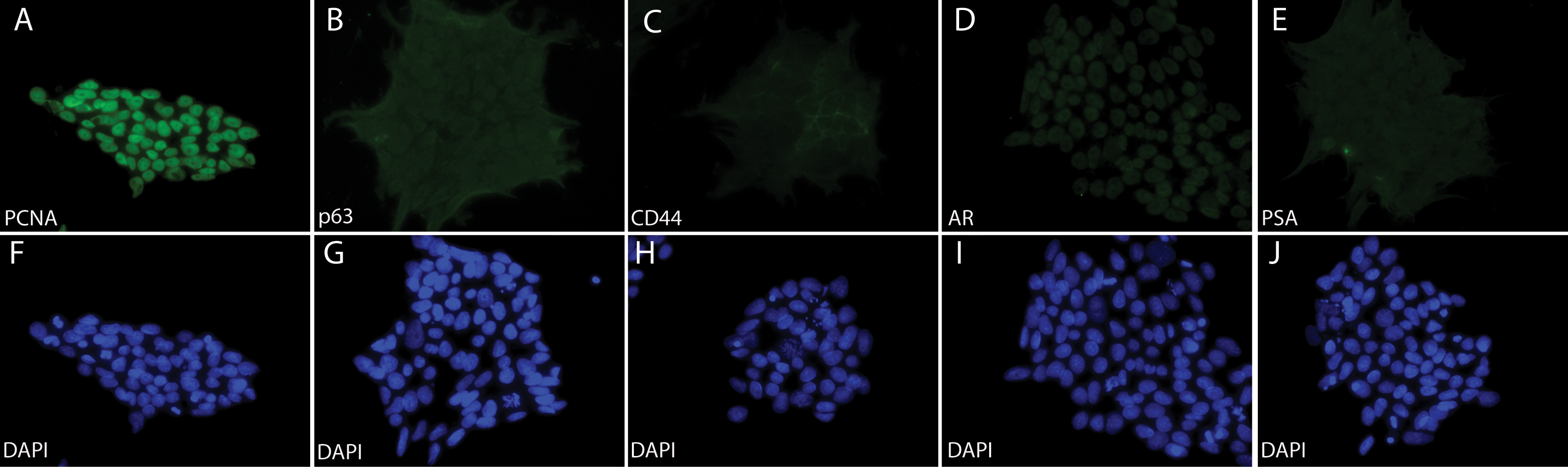

Supplement: Figure S2 — Immunofluorescence detection of marker expression in E-PZ-1-iPS-like-4 cells. E-PZ-1-iPS-like-4 cells showed strong nuclear staining of PCNA (A), which was used as a positive control. They did not express basal cell marker p63 (B), CD44 (C), AR (D), or PSA (E). (F), (G), (H), (I) and (J) are DAPI staining of the nuclei of the same cells in (A), (B), (C), (D), and (E), respectively. (TIF) [file pone.0064503.s002.tif]

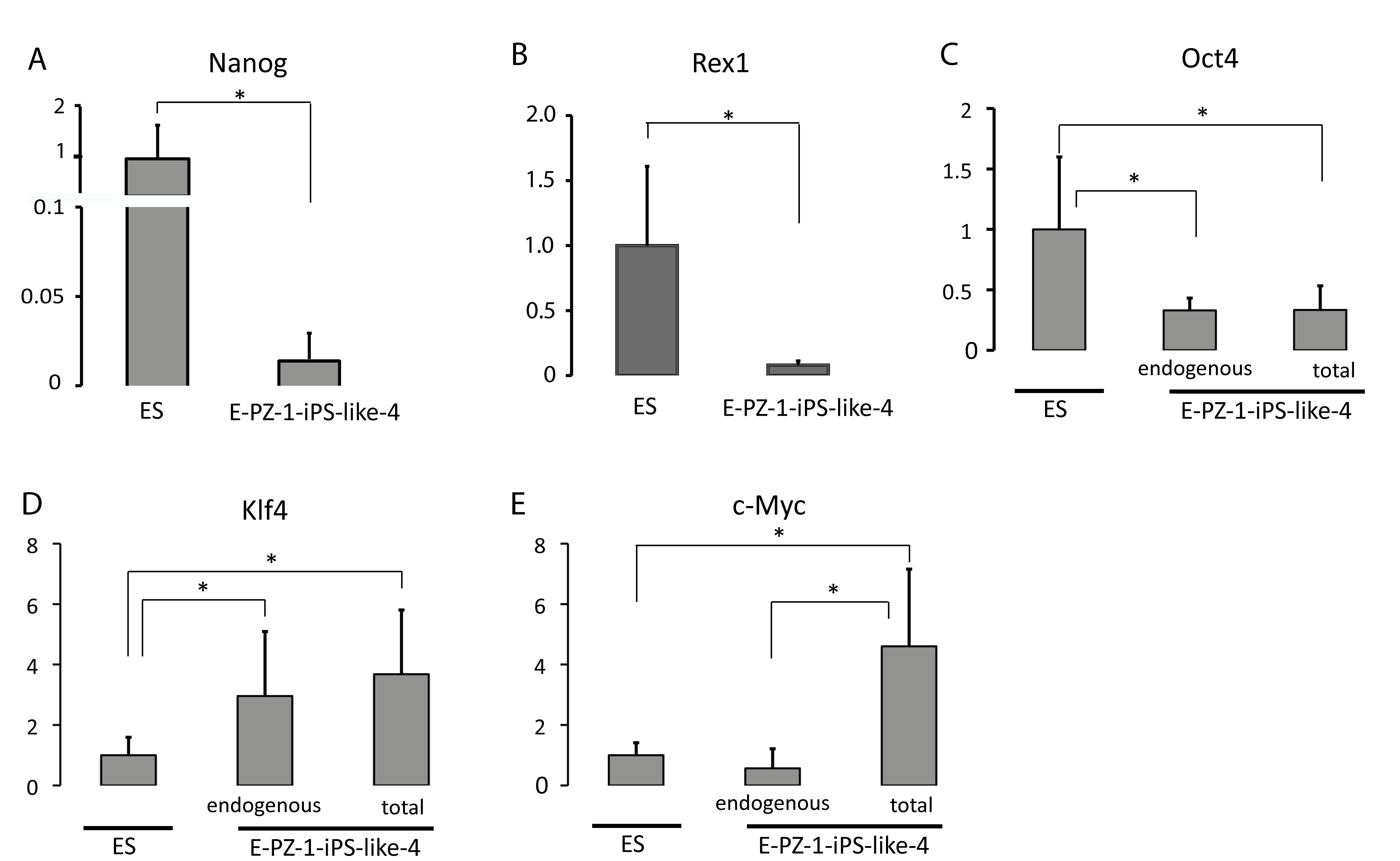

Supplement: Figure S3 — Comparison of expression levels of pluripotent genes in human ESCs (line H9) and E-PZ-1-iPS-like cells. mRNA levels of Nanog (A), Rex1 (B), total and endogenous Oct4 (C), total and endogenous Klf4 (D), and total and endogenous c-Myc (E) were measured by qRT-PCR and normalized against TBP. The Y-axis is the fold-level of gene expression in E-PZ-1-iPS-like cells compared to those in ES cells, which were set as 1. Asterisks indicate statistical significance by t-test. (TIF) [file pone.0064503.s003.tif]

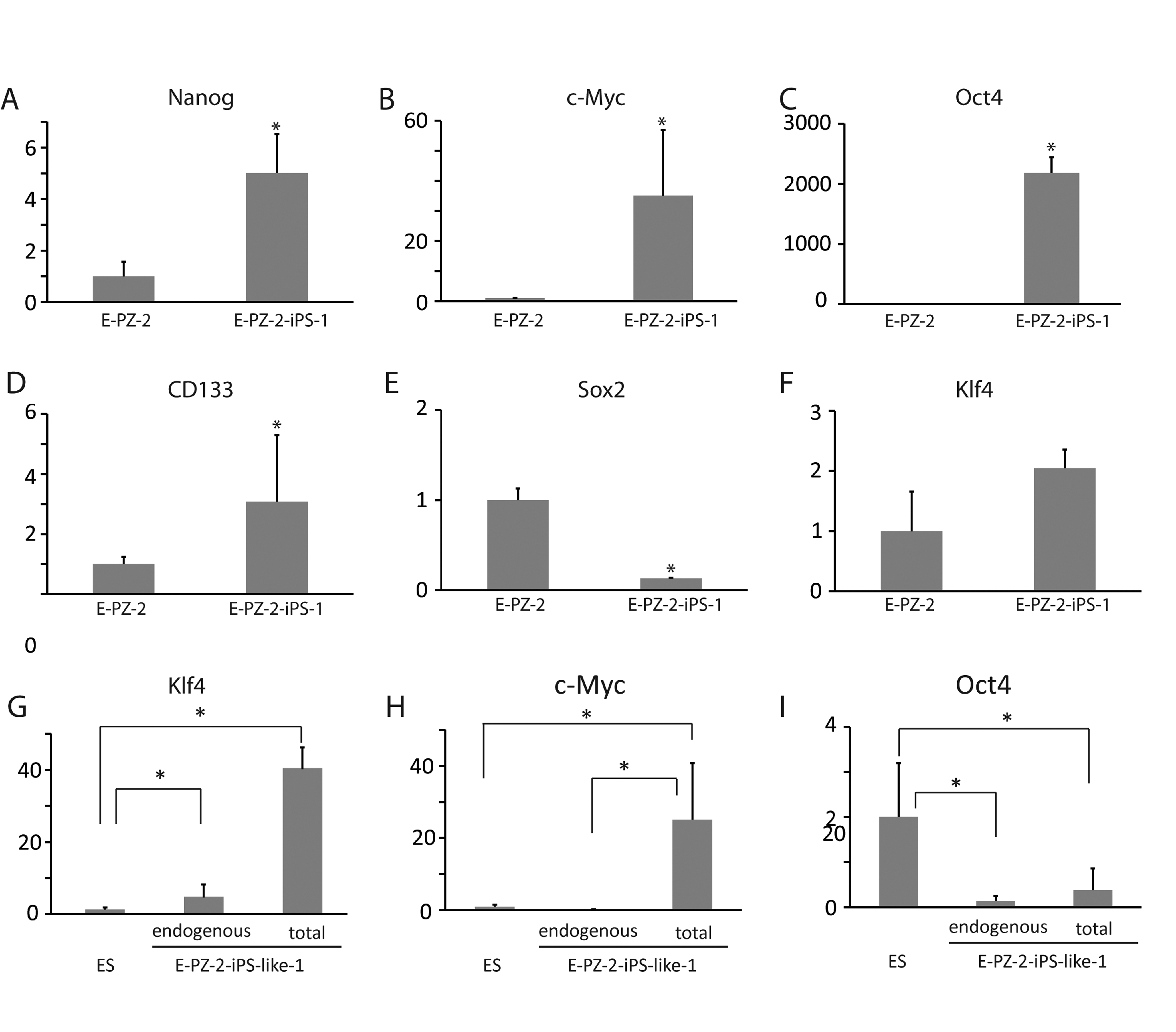

Supplement: Figure S4 — Expression levels of pluripotent genes in E-PZ-2-iPS-like-1 cells. mRNA levels of Nanog (A), total c-Myc (B), total Oct4 (C), CD133 (D), total Sox2 (E), and total Klf4 (F) in E-PZ-2-iPS-like-1 cells were compared to parent E-PZ-iPS-2 cells. Total and endogenous Klf4 (G), total and endogenous c-Myc (H), and total and endogenous Oct4 (I) were measured by qRT-PCR and normalized against TBP. In (A)–(F), the Y-axis is the fold-level of gene expression in E-PZ-2-iPS-like cells compared to those in E-PZ-2 cells, which were set as 1. In (G)–(I), the Y-axis is the fold-level of gene expression in E-PZ-2-iPS-like cells compared to those in ES cells, which were set as 1. Asterisks indicate statistical significance by t-test. (TIF) [file pone.0064503.s004.tif]

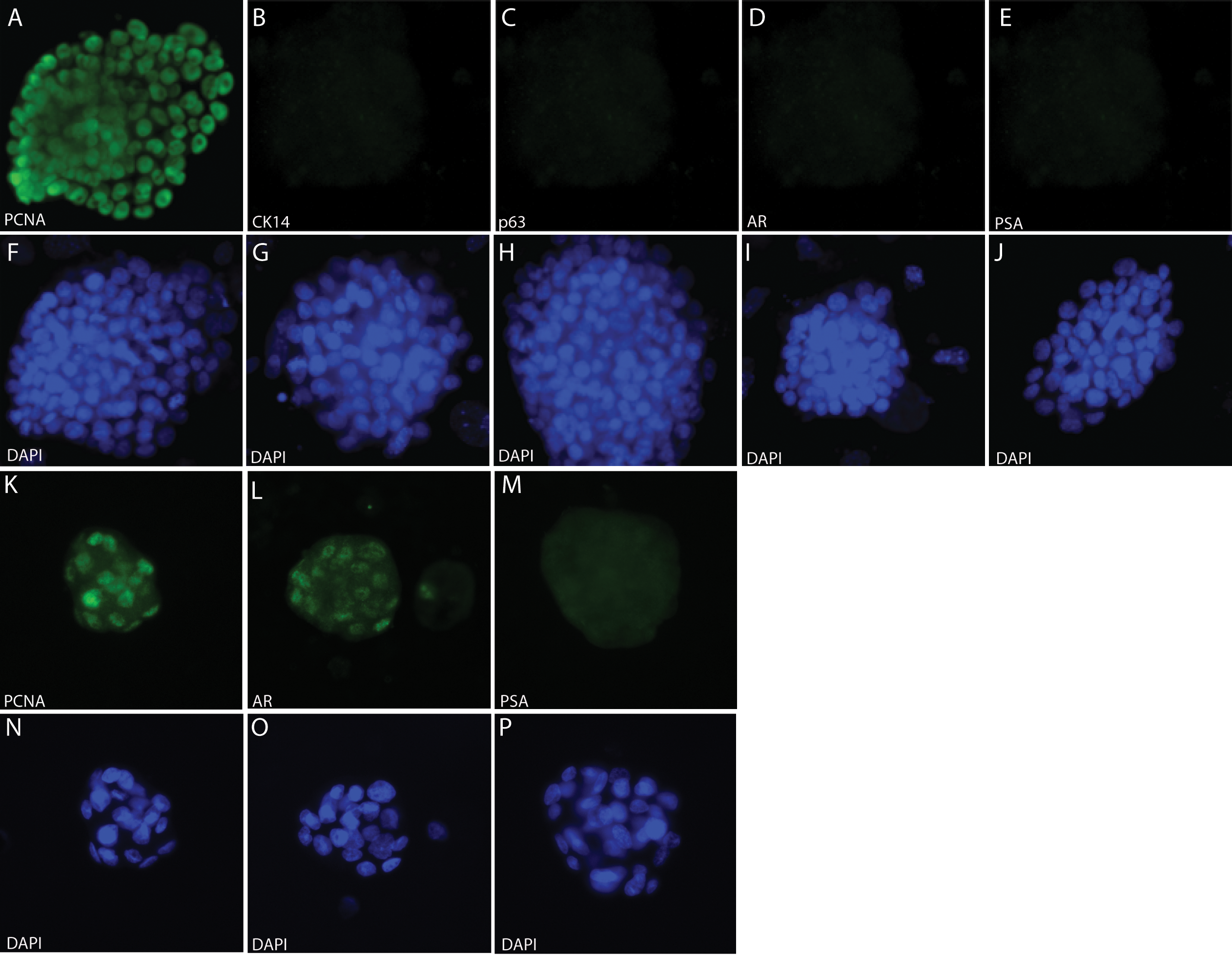

Supplement: Figure S5 — In vitro differentiation of F-iPS and E-PZ cells. F-iPS and E-PZ cells were subjected to conditions that induced differentiation of secretory prostatic epithelial cells, i.e., spheres were cultured in Complete PFMR-4A medium supplemented with 10 nM R1881 in the presence of rat UGS. An F-iPS-derived sphere showed strong staining of PCNA (A), but not CK14 (B), p63 (C), AR (D) or PSA (E). Spheres derived from E-PZ cells expressed PCNA (K). Some spheres also expressed an intermediate level of AR (L), but no PSA was detected (M). (F), (G), (H), (I), (J), (N), (O) and (P) are DAPI staining of the nuclei of the same cells in (A), (B), (C), (D), (E), (K), (L) and (M), respectively. (TIF) [file pone.0064503.s005.tif]

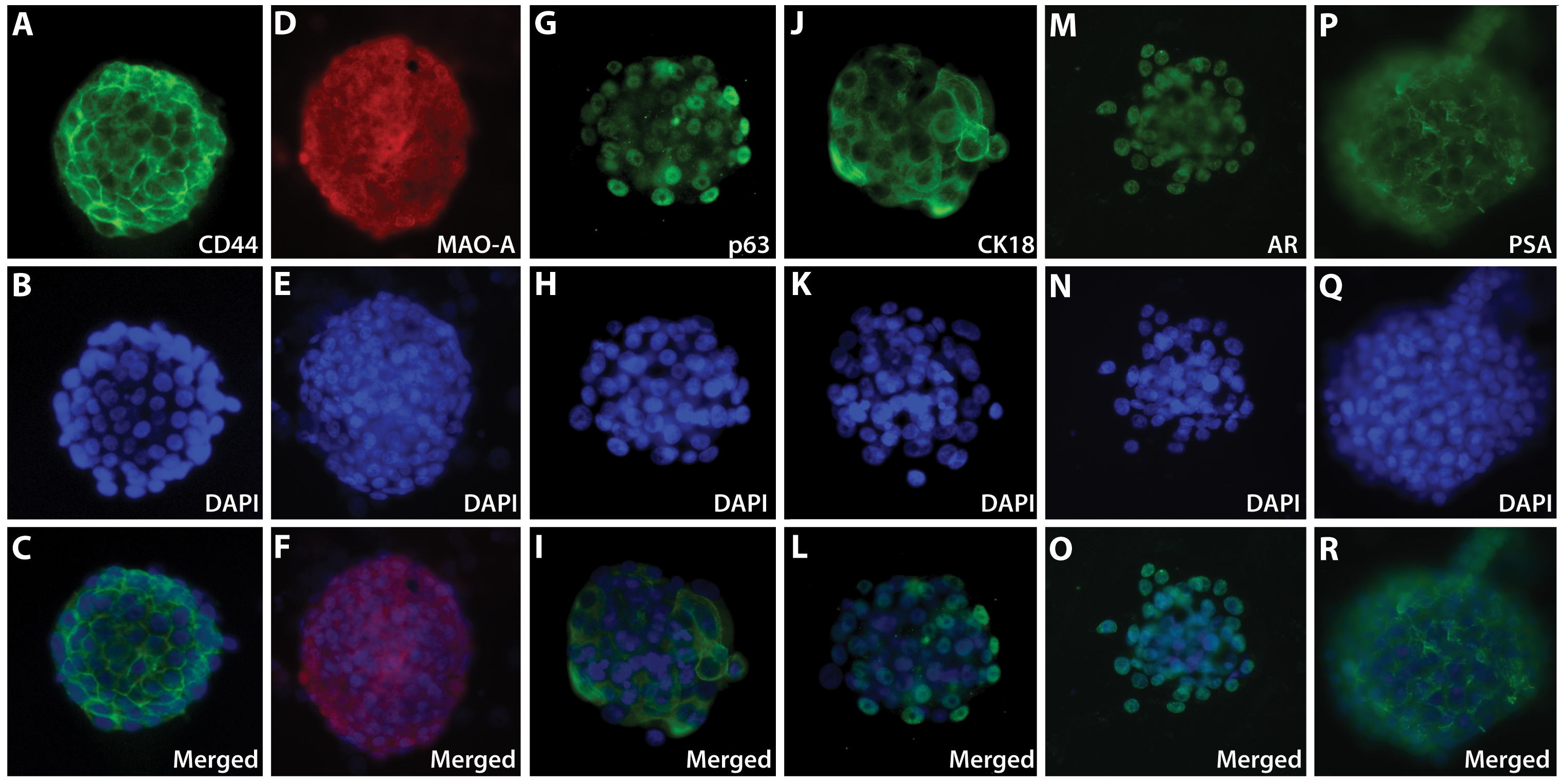

Supplement: Figure S6 — In vitro differentiation of E-PZ-2-iPS-like-1 cells. E-PZ-2-iPS-like-1 were cultured in E-PZ medium expressed basal prostatic epithelial cell markers including CD44 (A), MAO-A (D), and p63 (G). In addition, some spheres expressed CK18 (J) and AR (M) in the presence of R1881. When co-cultured with rat UGS, a subset of the spheres expressed PSA (P). (B), (E), (H), (K), (N) and (Q) are DAPI staining of the nuclei of the same cells in (A), (D), (G), (J), (M), and (P) respectively. (C), (F), (I), (L), (O), and (R) are merged images of (A) and (B), (D) and (E), (G) and (H), (J) and (K), (M) and (N), (P) ad (Q), respectively. (TIF) [file pone.0064503.s006.tif]

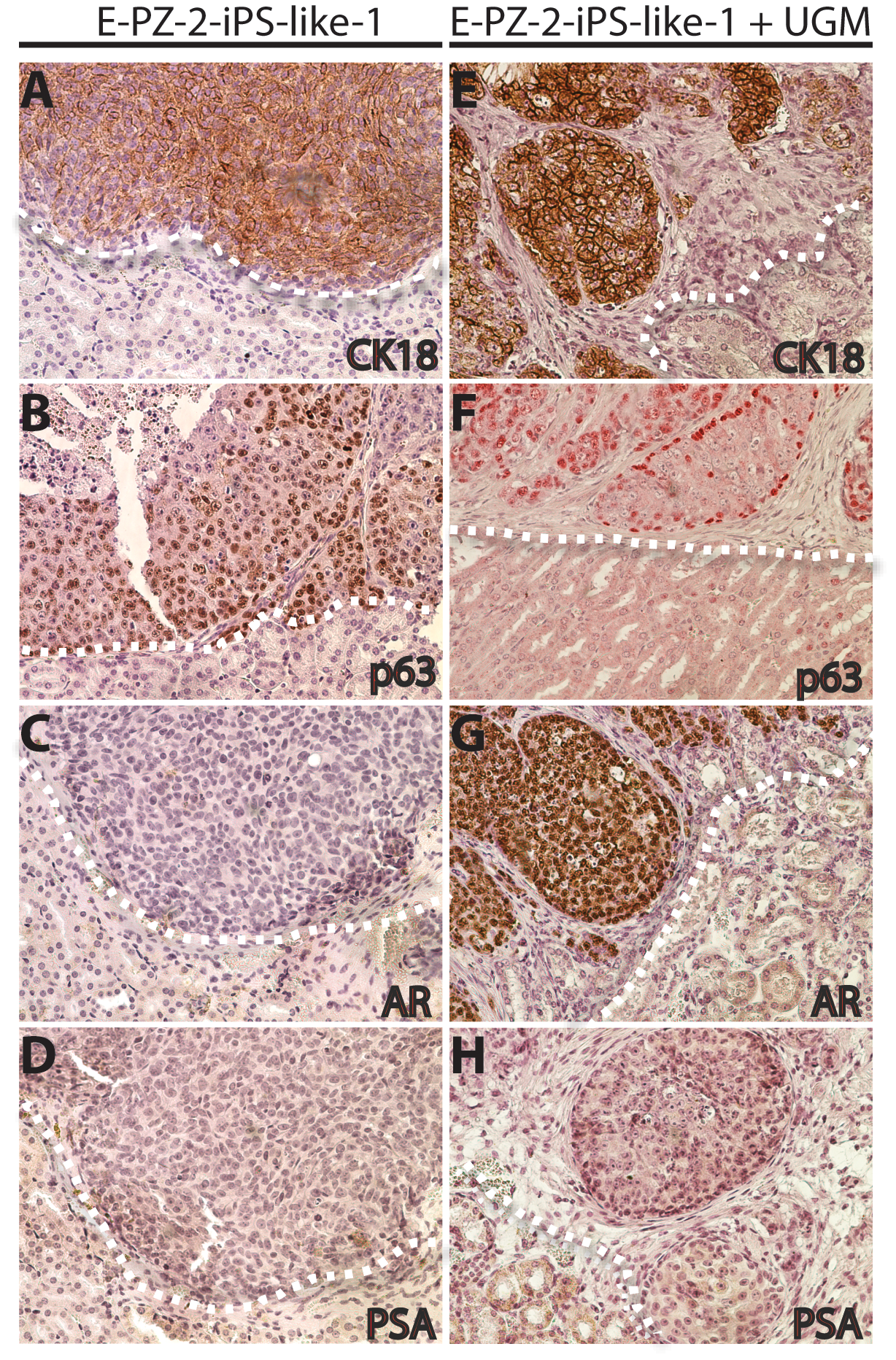

Supplement: Figure S7 — In vivo differentiation of E-PZ-2-iPS-like-1 cells. E-PZ-2-iPS-like-1 cells injected under the renal capsule of immunodeficient mice expressed basal prostatic epithelial markers p63 (B) and transit amplifying epithelial cell marker CK18 (A, but not the secretory cell markers AR (C) or PSA (D). When combined with UGM, E-PZ-2-iPS-like-1 cells gave rise to cell clusters that uniformly expressed CK18 (E), and p63 but only at the edge (F). Although the cells were negative for PSA (H), they expressed AR in the nuclei (G). White dotted lines mark the boundary of grafts derived from E-PZ-2-iPS-like-1 cells and mouse kidney. All magnifications are 20×. (TIF) [file pone.0064503.s007.tif]
