# Supplementary material for: Induced Pluripotency of Human Prostatic Epithelial Cells
Source: PLoS One. 2013 May 22;8(5):e64503. doi: 10.1371/journal.pone.0064503 (PMC3661502; doi:10.1371/journal.pone.0064503)
Supplement: Table S1 — Antibodies used in the study. (DOCX) [file pone.0064503.s008.docx]

| **Name** | **Source** | **Dilution** |
| --- | --- | --- |
| Anti-Oct3/4 | Santa Cruz Biotechnology, Santa Cruz, CA | 1:500 |
| Anti-Sox2 | Biolegend, San Diego, CA | 1:500 |
| Anti-c-Myc | Abcam, Cambridge, MA | 1:500 |
| Anti-SSEA-3 | Millipore, Billerica, MA | 1:500 |
| Anti-Tra-1–81 | Millipore | 1:500 |
| Anti-Ku70 | Abcam | 1:500 |
| Anti-Nanog | Abcam | 1:500 |
| Anti-MAP-2 | Leica Microsystems, Buffalo Grove, IL | 1:500 |
| Anti-Osteocalcin | R&D Systems, Minneapolis, MN | 1:200 |
| Anti-AFP | Abcam | 1:200 |
| Anti-MAO-A | Santa Cruz Biotechnology | 1:1000 |
| Anti-CK5 | Imegenex, San Diego, CA | 1:100 |
| Anti-cytokeratin 18 (CK18) | Santa Cruz Biotechnology | 1:200 |
| Anti-AR | BD Pharmingen, San Diego, CA | 1:200 |
| Anti-PSA | Santa Cruz Biotechnology | 1:100 |
| Anti-AMACR/p63 | Biocare, Concord, CA | ready to use |
| Anti-Ki67 | Biocare | 1:100 |
| Anti-PCNA | Santa Cruz Biotechnology | 1:100 |
| universal biotinylated horse anti-mouse/rabbit IgG | Vector Laboratories Inc., Burlingame, CA | 1:1000 |
| Alexa 488 goat anti-mouse | Invitrogen, Carlsbad, CA | 1:1000 |
| Alexa 555 goat anti-rabbit | Invitrogen | 1:1000 |

Table S1 Antibodies used in the study
