# Supplementary material for: Induced Pluripotency of Human Prostatic Epithelial Cells
Source: PLoS One. 2013 May 22;8(5):e64503. doi: 10.1371/journal.pone.0064503 (PMC3661502; doi:10.1371/journal.pone.0064503)
Supplement: Table S2 — Primer sequences used in the study. (DOCX) [file pone.0064503.s009.docx]

Table S2 Primer sequences used in the study.

| **Gene** | **Direction** | **Sequence** |
| --- | --- | --- |
| *total Oct4* | forward | AGCGAACCAGTATCGAGAAC |
|  | reverse | TTACAGAACCACACTCGGAC |
| *endogenous Oct4* | forward | CCTCACTTCACTGCACTGG |
|  | reverse | CAGGTTTTCTTTCCCTAGCT |
| *Nanog* | forward | TGAACCTCAGCTACAAACAG |
|  | reverse | TGGTGGTAGGAAGAGTAAAG |
| *Sox2* | forward | AGCTACAGCATGATGCAGGA |
|  | reverse | GGTCATGGAGTTGTACTGCA |
| *Rex1* | forward | TGACAAAGGGGACGAAGCAAGAG |
|  | reverse | GCCATCAAAAGGACACACAAAG |
| *total c-Myc* | forward | ACTCTGAGGAGGAACAAGAA |
|  | reverse | TGGAGACGTGGCACCTCTT |
| *endogenous c-Myc* | forward | TGCCTCAAATTGGACTTTGG |
|  | reverse | GATTGAAATTCTGTGTAACTGC |
| *total Klf4* | forward | TCTCAAGGCACACCTGCGAA |
|  | reverse | TAGTGCCTGGTCAGTTCATC |
| *endogenous Klf4* | forward | GATGAAACTGACCAGGCACTA |
|  | reverse | GTGGGTCATATCCACTGTCT |
| *CD133* | forward | TCAGTGAGAAAGTGGCATCG |
|  | reverse | GCTTTTCCTATGCCAAACCA |
| *TBP* | forward | TGCTGAGAAGAG TGTGCTGGAG |
|  | reverse | TCTGAATAGGCTGTG GGGTC |
